# Supplementary figures and images for: Repetitive training enhances the pattern recognition capability of cultured neural networks
Source: PLoS Comput Biol. 2025 Apr 22;21(4):e1013043. doi: 10.1371/journal.pcbi.1013043 (PMC12064202; doi:10.1371/journal.pcbi.1013043)

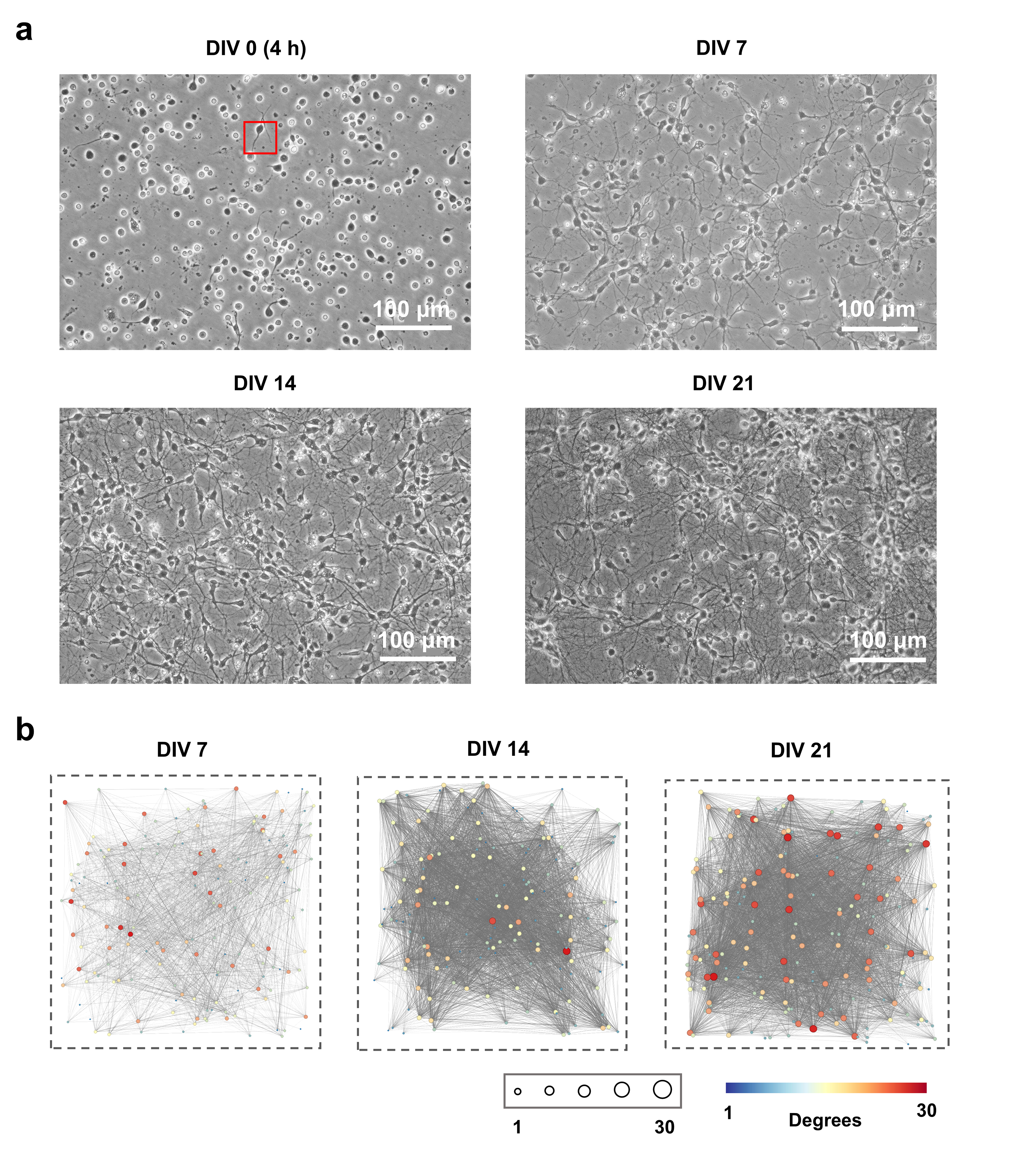

Supplement: S1 Fig — (a) Structural changes of neurons cultured in vitro at different developmental time points. (b) Functional network connectivity, where nodes represent neurons, node size represents node degree, and the larger the node degree, the color is close to red, and the lower is close to blue. (TIF) [file pcbi.1013043.s001.tif]

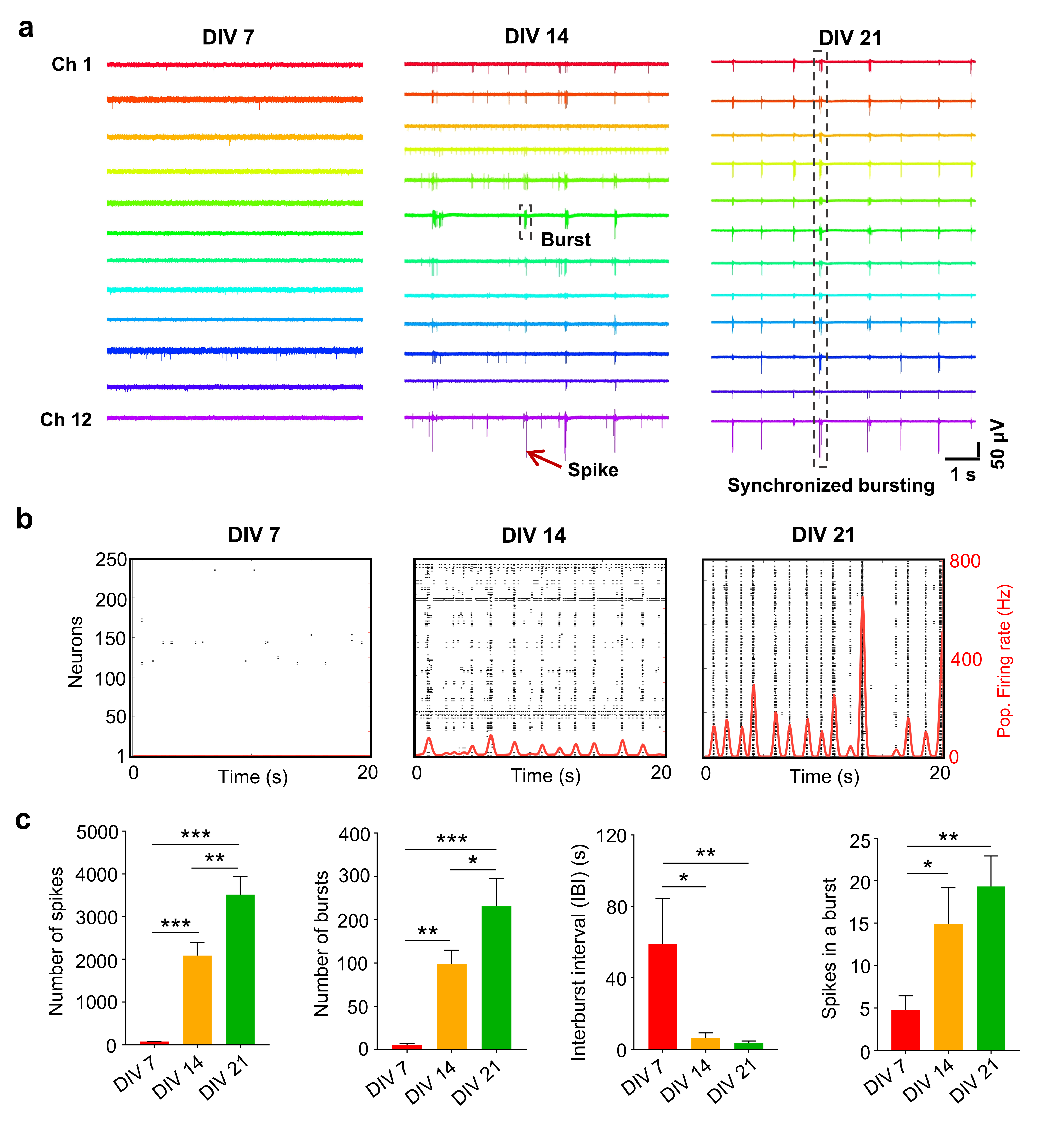

Supplement: S2 Fig — (a) Original waveform graph of spontaneous electrical activity in neural networks at different developmental time points. (b) Raster plot of spontaneous activity in neural networks, where the black lines represent a single spike, and the red lines represent the firing rate of the neuronal network. (c) Quantitative indicators of activity in neural networks cultured in vitro at different developmental days (Number of spikes; number of bursts; interburst interval, IBI; spikes in a burst) (mean±s.m.e., n = 5, *p < 0.05, **p < 0.01, ***p < 0.001). (TIF) [file pcbi.1013043.s002.tif]

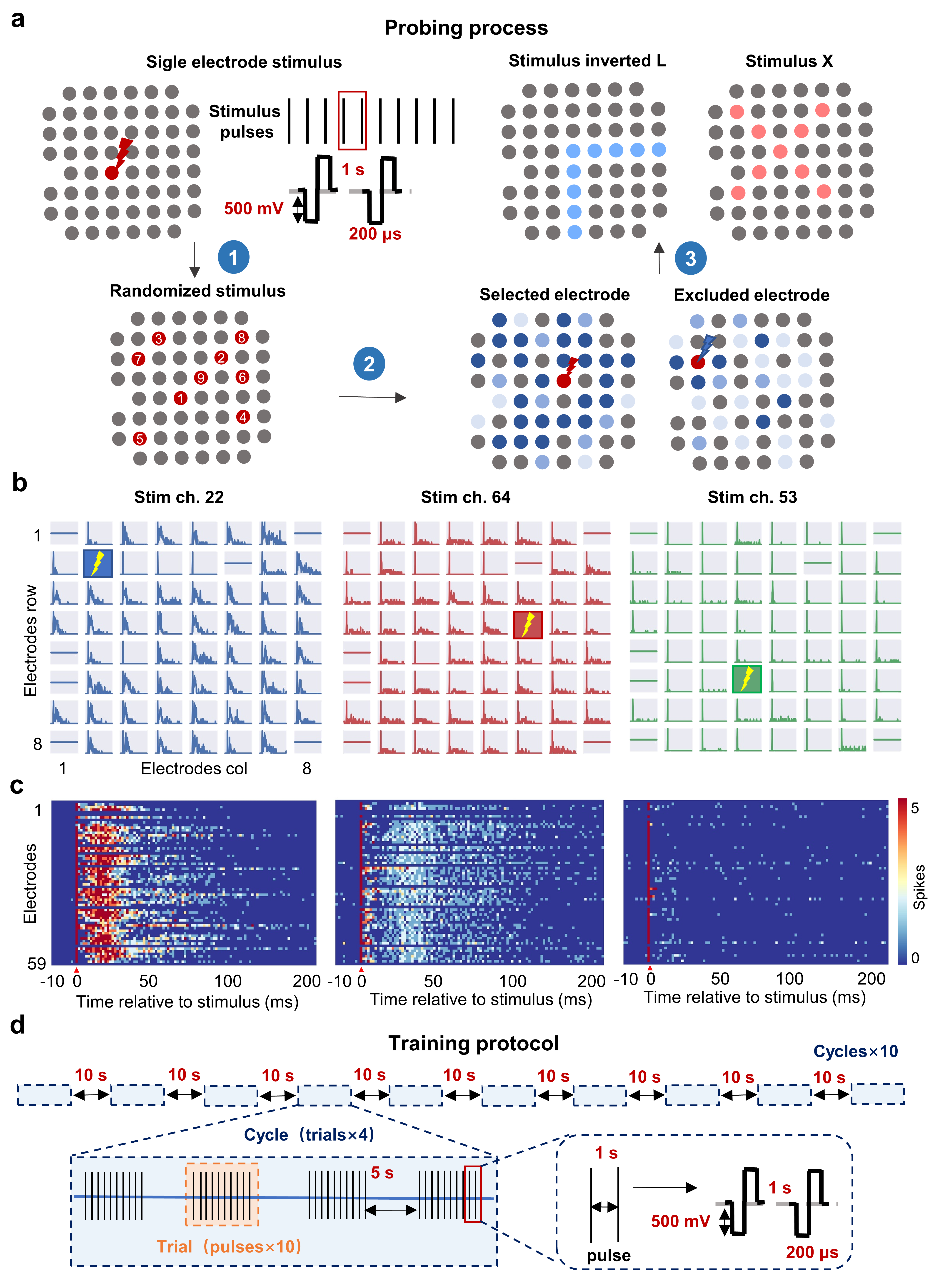

Supplement: S3 Fig — (a) Select stimulation points and combine them into a stimulation pattern. (b) PSTH (Peri-Stimulus Time Histogram) response of neural networks induced by different stimulation. The higher the induced response intensity, the larger the PSTH area. (c) In the corresponding response activity diagram after stimulation in (b), the stronger the response, the closer to red; the weaker the response, the closer to blue. (d) Specific training stimulation paradigm. (TIF) [file pcbi.1013043.s003.tif]

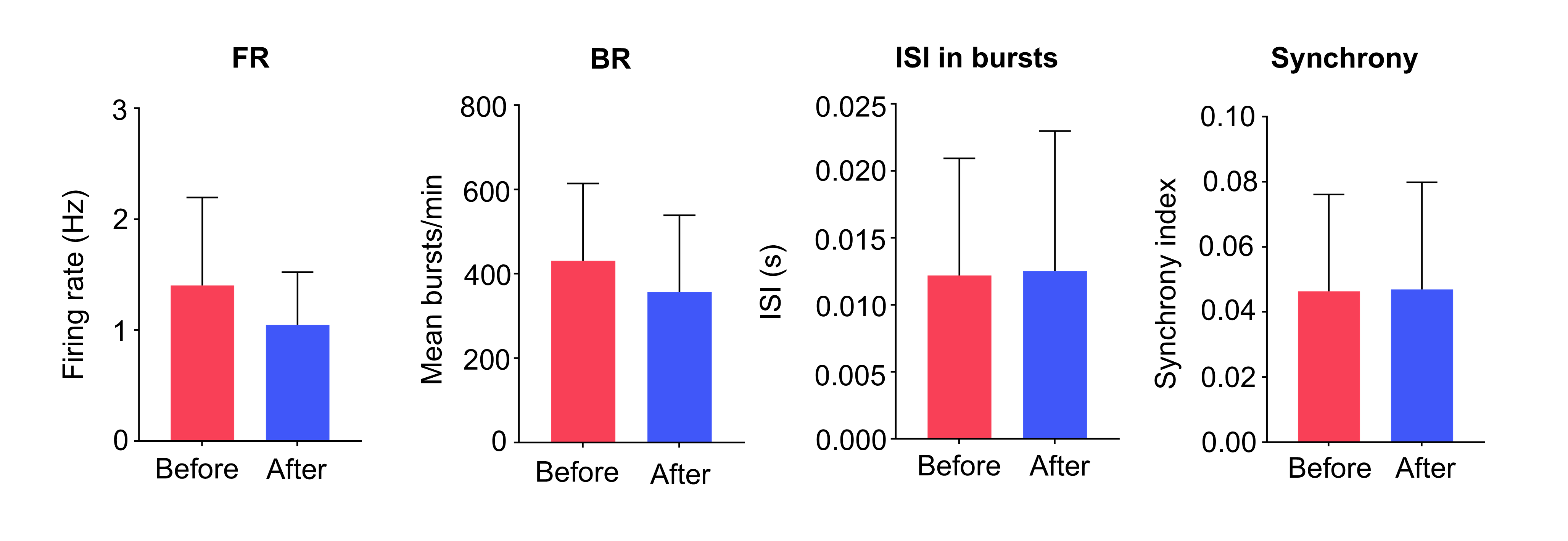

Supplement: S4 Fig — A comparative analysis was conducted about the spontaneous neural activity of the untrained control group. The control group was subjected to electrophysiological signal acquisition at intervals identical to those of the trained group, albeit in the absence of any stimulus-based training regimen. During data analysis, the control group was maintained under equivalent experimental conditions, encompassing an identical neurodevelopmental temporal framework. (TIF) [file pcbi.1013043.s004.tif]

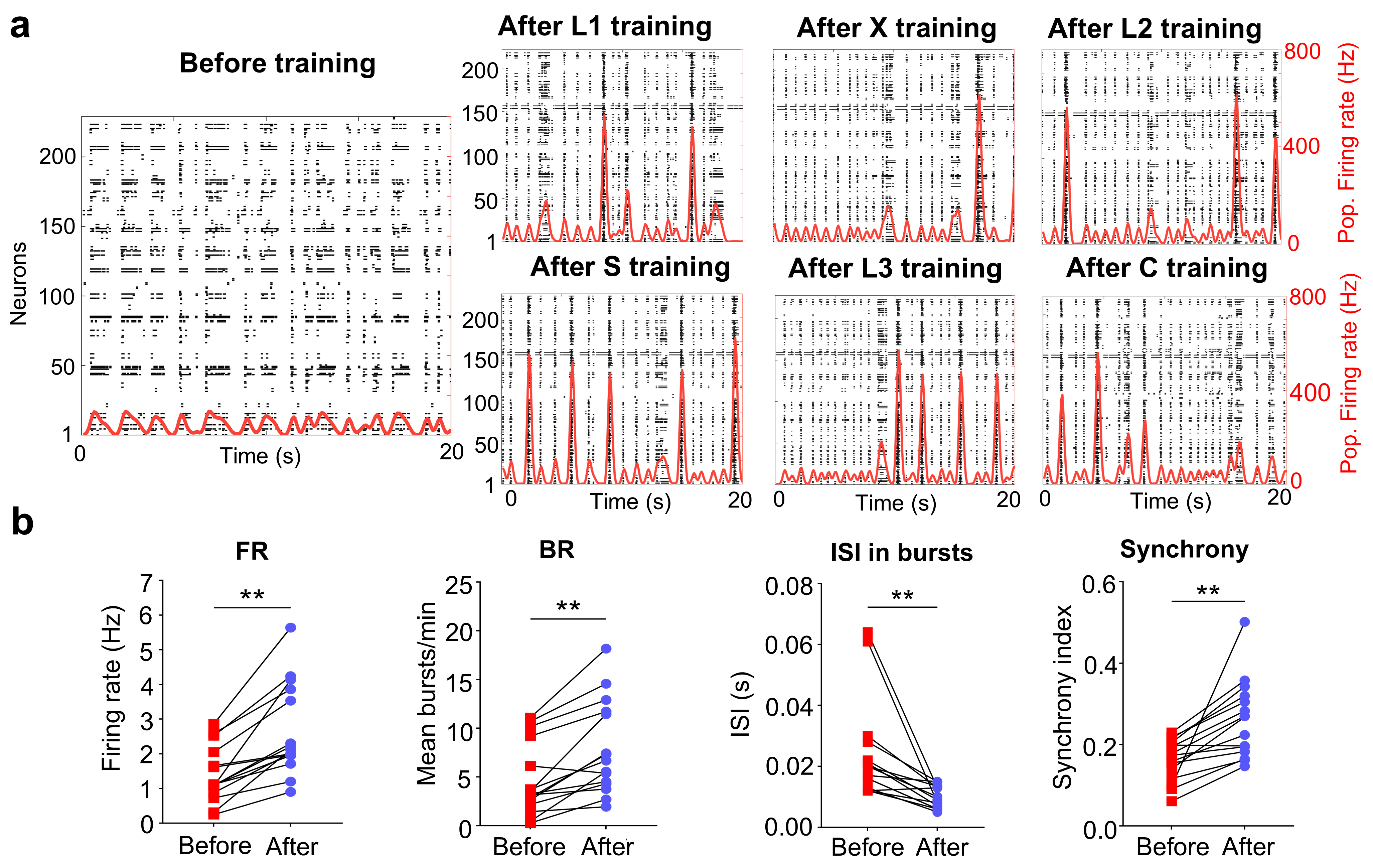

Supplement: S5 Fig — (a) Raster plot of spontaneous activity in neural networks, where the black lines represent a single spike, and the red lines represent the firing rate of the neuronal network. (b) Quantitative indicators of neuronal activity before and after training (mean firing rate, MFR; mean bursting rate, MBR; interburst interval, IBI; interspike interval (ISI) in a burst; synchrony index) (n = 10, paired t-test, *p < 0.05, **p < 0.01, ***p < 0.001). (TIF) [file pcbi.1013043.s005.tif]

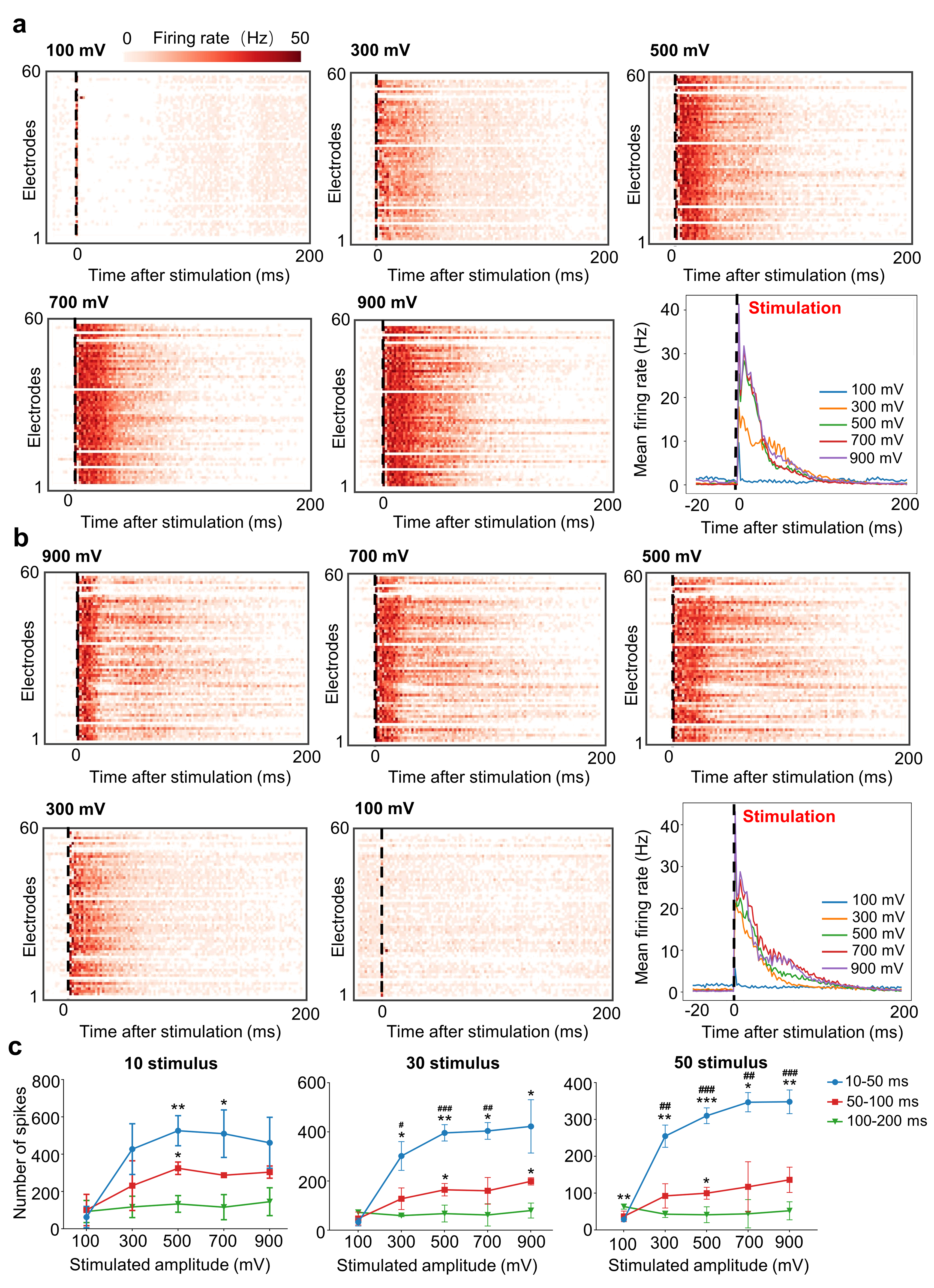

Supplement: S6 Fig — (a) Neuronal network activity is induced in ascending order of amplitude; the darker the color, the stronger the evoked firing rate. The lower right corner displays the average discharge rate of the neuronal network under different amplitude stimulations (b) In contrast to the stimulus amplitude order in (a). (c) The induced firing rate of the neuronal network under different numbers of stimulus and different amplitude stimulations (1Hz). (mean±m.s.e., n = 5). (TIF) [file pcbi.1013043.s006.tif]

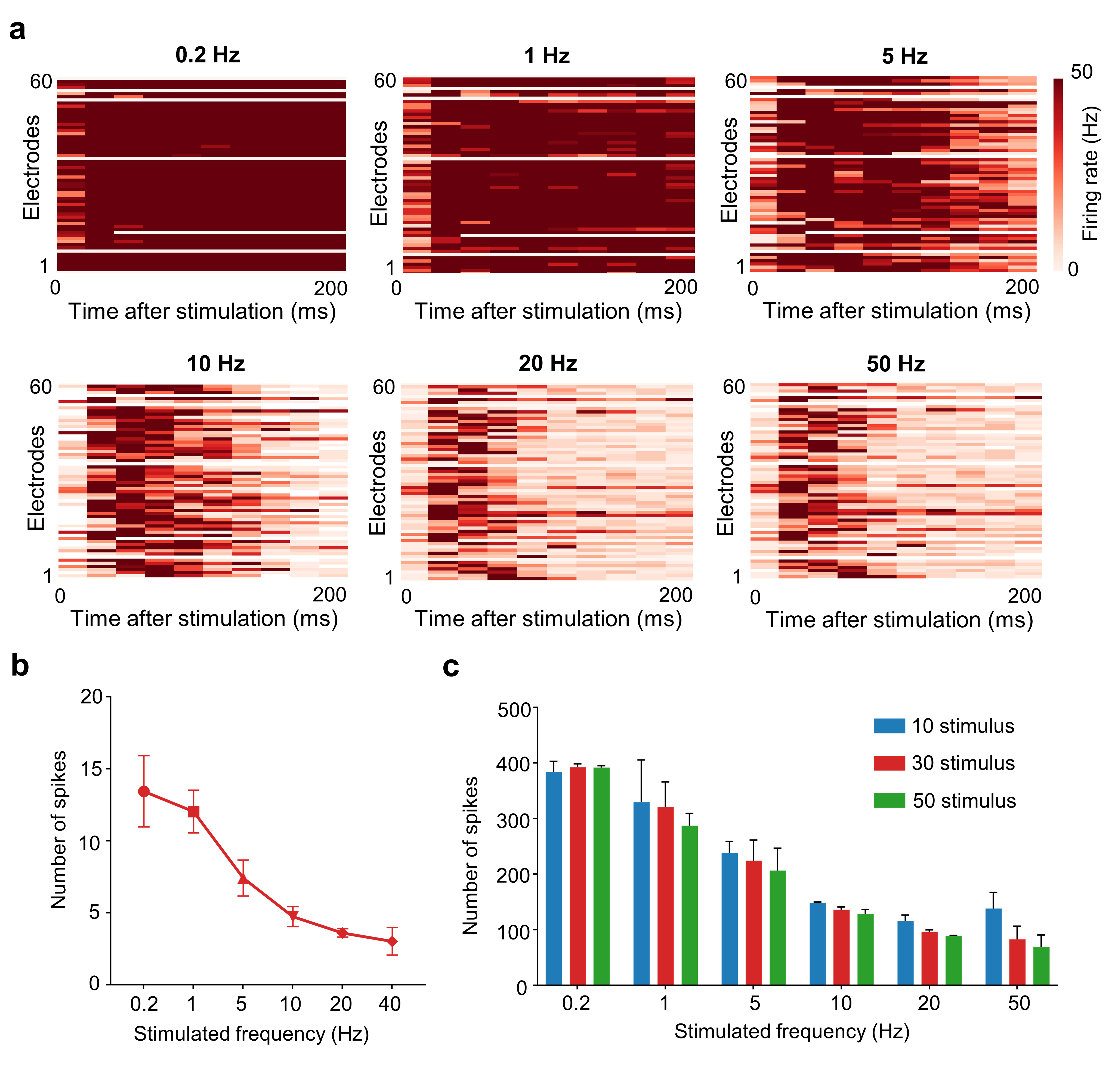

Supplement: S7 Fig — (a) Different stimulation frequencies induce activity in the neural network. (b) The evoked response of neural networks under different frequency stimulation was compared. (c) Different stimulus frequencies correspond to the evoked response under different stimulus. (TIF) [file pcbi.1013043.s007.tif]

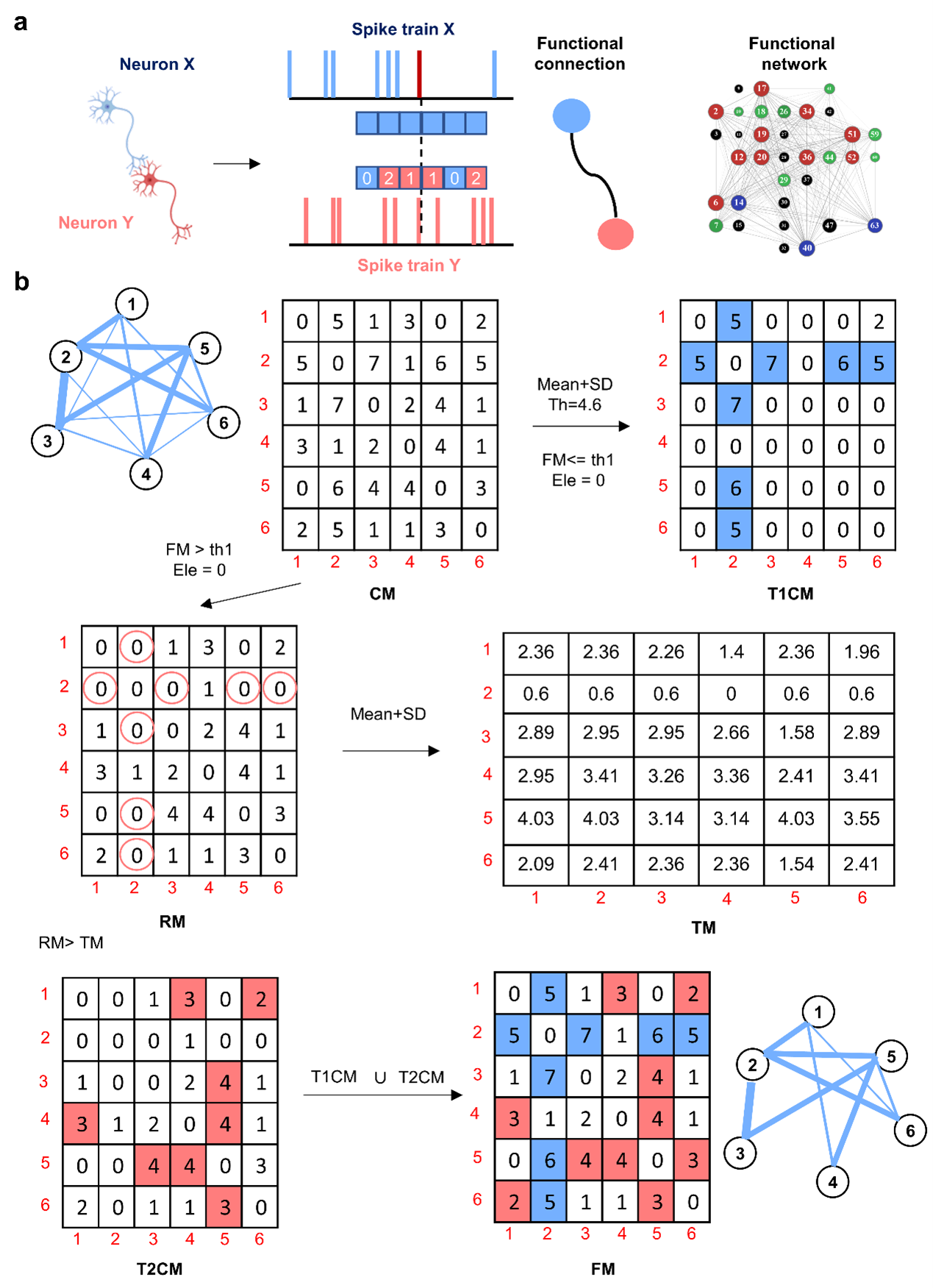

Supplement: S8 Fig — (a) The cross-correlation algorithm is used to calculate the correlation between the spike train of two neurons as the edge weight. (b) Double Threshold (DDT) algorithm operating principle applied to a simple network. (TIF) [file pcbi.1013043.s008.tif]
