# Supplementary material for: Repetitive training enhances the pattern recognition capability of cultured neural networks
Source: PLoS Comput Biol. 2025 Apr 22;21(4):e1013043. doi: 10.1371/journal.pcbi.1013043 (PMC12064202; doi:10.1371/journal.pcbi.1013043)
Supplement: Attachment — Submitted filename: Comments to the authors.pdf [file pcbi.1013043.s009.pdf]

Revision manuscript "Repetitive training enhances the pattern recognition capability of cultured neural networks".

Summary:

The authors use cortical neural networks from rodents plated on multi-electrode arrays (MEAs). These networks are subjected to different electrical stimulation sequences, and electrophysiological activity is recorded before, during, and after the stimulation period. The main goals are to investigate these networks' stimuli classification ability and the mechanisms involved. To perform this study, the authors mainly refer to connectivity analysis and network burst activity.

Even though the manuscript presents very interesting results regarding the effects of external repeated stimuli on the network structure and the fact that different stimuli induce clearly different responses, at the current stage, it still needs clarifications and improvements. My main concern regards the number of experiments performed.

Major comments:

1- I find the research intriguing, and the results on connectivity analysis are indeed in line with previous work aimed at investigating memory in-vitro. My main concern, though, is the number of cultures used for the final analysis. In each figure, the authors mention that n=5. Could the authors explain why such a low number of cultures was used? Because, in general, the number of cultures used in these studies, when on single MEAs (60 electrodes), is usually around 10 (see Dias et al. 2020, Lamberti et al. 2024), I am wondering if the authors performed any power analysis demonstrating that 5 cultures would be enough for statistical analysis interpretation.
2- In their manuscript, one of the main findings is that after stimulation, not only do pre-existing connections change in strength but there is also the formation of new connections in the network (see line 155). How would the authors explain such a mechanism? In addition, although in methods, the authors mention the cultures that were used after 21 DIV, in the caption of Figure 1, the authors talk about cultures at DIV 14. Were the experiments performed at DIV 21 or DIV 14? I believe a clarification is needed. Cultures need 21 DIV to reach full maturation, so if experiments were performed before that, the creation of new connections might be due to the still ongoing network growth and not to the given stimulation.
3- As far as I understand, the authors use analysis on connectivity as the main readout. I would suggest already introducing in the introduction the importance of studying changes in connections to investigate memory and, consequently, information processing and storage by the networks. This will help with readability and will give input on why that was chosen as a metric.
4- In the data availability statement, the authors declare that no data were used for the study. What is meant by this? Weren't recordings of neuronal activity performed and analyzed for the study? If yes, why are the authors not sharing data on an online data repository or upon request?

Minor comments:

1- In the methods section, under "Burst and network burst detection" in line 408, the authors wrote that if at least 20% of the channels were recording simultaneous activity, that was defined as a network burst. Can the authors better clarify why the threshold on

the amount of electrodes was set to 20%? The definition and analysis of network bursts in MEAs is an ongoing debate; it will improve the strength of the manuscript by providing more details on how network bursts were identified, also given that it is one of the readouts used to interpret results.

2- In the methods section under " Functional network construction and evaluation," lines 446-447, the authors say that a widely used cross-correlation algorithm was adopted. Can the authors provide references to the works in which this algorithm was already used for analysis? In addition, in line 449, the authors refer to the spiking activity of 2 neurons. Were the authors applying any spike sorting algorithm? How were they capable of differentiating single neurons from the MEAs recording? In MEA (60 electrodes) recordings, electrodes acquire activity from groups of neurons and not single neurons, so if no spike sorting was applied probably it the sentence should change to "spike activity of 2 electrodes".

3- The same goes for the Euclidean distances section; also, here, the authors say that it is a common method used to analyze changes in functional network structure but provide only one reference. Providing more references to similar work using the same method would strengthen the statement.

4- References in lines 459 and 480 are in a different format compared to the rest of the manuscript.

5- Typo line 435 "… and The PSTH..". The T in the should not be capitalized.
