# Supplementary material for: Repetitive training enhances the pattern recognition capability of cultured neural networks
Source: PLoS Comput Biol. 2025 Apr 22;21(4):e1013043. doi: 10.1371/journal.pcbi.1013043 (PMC12064202; doi:10.1371/journal.pcbi.1013043)
Supplement: Attachment — Submitted filename: 0226 response-PCOMPBIOL-D-24-01467_2_13.docx [file pcbi.1013043.s011.docx]

**Responds to the reviewers’ comments**

Reviewer #1 (Remarks to the Author):

This work studies the effect of training on culture networks and the association between evoked and spontaneous activity by looking at various properties of neural and network activity. The topic is interesting and experiments are meaningful. There are a few issues related to the presentation and design of the work. Hope the comments below are constructive.

**Response:** We sincerely thank your valuable feedback that we have used to improve the quality of our manuscript. Your comments are laid out below and specific concerns have been numbered. Our response is given in blue text, and changes/additions to the manuscript are provided in red.

1. One major point is that extracting spikes from the MEA data could have some problems. Using a threshold cutoff for spikes was not proper to separate different neurons. It is necessary to do spike sorting to have better the quality of neurons. Otherwise, they are more like MEA activity rather than single neurons. As such, functional network connectivity could have prudential issues. Since the data was collected, could be good to do spike sorting and get a better resolution of single neurons. Given the focus here is to study underlying neuronal mechanisms, using a better way of spike sorting is necessary.

**Response**: We agree with the reviewer and have used the spike sorting-SpyKING CIRCUS algorithm (*Yger et al., Elife. 2018,7:e34518; Lee et al.,* *Neuroscience. 2021, 458:108-119; Hudetz et al., Front Neurosci. 2024, 18:1387098*) to re-analyze the data. Accordingly, the functional connectivity network is now constructed based on the spike data of single neuron. Spike sorting processes are detailed in the Methods section. The results demonstrated that new analysis method did not change our main conclusions, such as pattern recognition ability and network structure differences between same pattern stimulation or different pattern stimulation. However, we observed that in revised Figure 6a, when applying spike sorting algorithms to examine the alterations in the evoked neural network structure, the results indicated that the average degree and density of the functional networks across the six patterns did not exhibit significant changes with respect to the number of training days. This suggests that metrics such as network density do not account for the mechanism underlying the observed improvement in classification accuracy as the training period progresses. In our subsequent analyses, the comparative results of the functional network structures under same or different stimulation patterns will be essential for elucidating this mechanism. In revised Figure 1e-g, Figure 2, Figure 3, Figure 4c-d, Figure 6, Figure 7, and supplementary figures (Figure S1, Figure S2, Figure S4 and Figure S5), spike sorting was adopted.

2. When comparing the spontaneous activity of the network before and after training, could be better to have control cases, where there is no training applied but have the same time interval (say a few hours similar to the training time) of neural development. It is well known that neuronal tissue can develop quickly to generate essential network activity.

**Response**: Thank you for your insightful comment. We appreciate the suggestion to consider the natural development of neural tissue over time. In response, we conducted additional experiments comparing spontaneous activity in control groups that were subjected to the same time interval as the training group, but without any applied training. These control groups were exposed to identical experimental conditions, including the same time window for neural development.

The results indicate that, in the untrained control group, analysis of spontaneous electrical activity over the same time period revealed no significant changes (revised supplementary Figure 4). This control experiment confirms that the alterations in spontaneous electrical activity observed in the trained experimental group were indeed attributable to the applied stimulation, rather than reflecting intrinsic developmental changes in the neural network.

3. The abstract could be better revised. “cultured neural networks in vitro have demonstrated biological intelligence” sounds strange. Cultured tissues are biological Intelligence was used in many locations but without proper definition. Classification patterns are not intelligence. The study here is to classify the neuronal pattern given different stimuli.

**Response**: Thank you for the suggestion. We have revised the manuscript by removing the previous discussion of biological intelligence in relation to cultured neural networks and replacing it with a more focused description of tasks: “Cultured neural networks in vitro have demonstrated the biocomputing capability to recognize patterns. However, the underlying mechanisms behind information processing and pattern recognition remain less understood.” , “This work provides new insights into the structural changes underlying information processing and contributes to our understanding of how cultured neural networks respond to different patterns. ”

4. There are many places with overstated claims. Particularly, the text about AI is overstated, while the focus here is only related to playing cultured networks with various stimulations.

**Response**: We have removed all connections to AI and revised our introduction: “In recent years, there has been growing interest in the biological computation capabilities of *in vitro* cultured neural networks. These networks are increasingly recognized as valuable platforms for investigating fundamental neural processes.”

5. When using six stimulations, it may be necessary to have other cases for comparison. For example, the same order and different orders, or random order of stimulation.

**Response**: Thank you for your insightful suggestion. We fully appreciate your concerns regarding the sequence of training with different patterns. We carefully examined potential concerns related to the order of stimulation. One concern might be that the cultured neural network could recognize only a specific sequence of pattern groups, rather than independently recognizing each individual pattern. However, our training paradigm involves repeating each pattern 400 times, followed by a 10-minute rest before transitioning to the next pattern, rather than cycling through all six patterns sequentially. This design ensures that the training for each pattern is independent, with no sequential dependency. Another concern pertains to the possibility that the order of training might affect recognition performance, specifically whether patterns trained later may exhibit lower recognition accuracy. To better address your concern and further investigate whether the training order is related to the neural network's ability to recognize sequential information, we analyzed the classification accuracy for four patterns and compared these results with those from the first four patterns, as shown in Figure 4. The trends were consistent (accompanying table 1), with no statistically significant differences, indicating that the order of pattern stimulation does not influence recognition performance following training.

Accompanying table 1

|  |  | In normal order | In reverse order |
| --- | --- | --- | --- |
| Cell1 | TD1 | 0.89 | 0.845 |
|  | TD2 | 0.91 | 0.935 |
|  | TD3 | 0.94 | 0.95 |
| Cell2 | TD1 | 0.81 | 0.772 |
|  | TD2 | 0.86 | 0.81 |
|  | TD3 | 0.89 | 0.83 |
| Cell3 | TD1 | 0.745 | 0.755 |
|  | TD2 | 0.749 | 0.743 |
|  | TD3 | 0.812 | 0.792 |

Moreover, we reviewed a comparable study on pattern stimulation, which involved speech recognition training on brain organoids conducted over two training days. In that study, the order of stimulation patterns was not addressed as having any impact on the conclusions drawn after training (*Cai et al., Nat Electron, 2023, 1032-1039*). Furthermore, we considered whether training on one pattern could influence the response to subsequent patterns. In revised Supplementary Figure 6, we examined the responses to varying stimulation amplitude gradients. Two stimulation sequences were tested: from low to high and from high to low. The results revealed that the order of stimulation patterns did not affect the neural network's responses.

6. Citing references should have the same format: sometimes using 1,2 and 3, but sometimes using Yang et al.,2023 at line 67.

**Response**: Thanks for your careful suggestion. We have corrected it to the numerical format and rechecked all the format of citing references.

7. Fig 1b: the authors state that both activities are similar at line 107. It is not very clear how they are similar. Maybe it is necessary to add a few characteristics for both signals.

**Response:** Thanks for the thoughtful suggestion. We realize that our description was unclear and requires correction. The signals in Fig. 1b represent two different types of neuronal activities: the top panel shows representative spontaneous neuronal activity, while the bottom panel represents evoked neuronal activity induced by stimulation. These two signals are not directly similar but rather reflect neuronal electrophysiological function. Fig. 1b is a schematic for spontaneous and evoked neuronal activity, not intended to analyze the characteristic differences between these two signals.

We have rewriten the description of Figure 1b: “Specifically, we clarified that only when the neuronal network reaches mature development, characterized by spontaneous activity and the ability to produce evoked responses upon stimulation, are the conditions met for conducting experiments (Figure 1b). This criterion ensures the reliability and relevance of the experimental data.”

8. Fig 1c is not clear: what is the green period for?

**Response**: Thanks for the comments. Fig 1c shows the two-pattern stimulation (training) protocol. The green period represents the probing phase, when the stimulation electrodes for the patterns are selected before training. Candidate stimulation electrodes are determined based on the criterion that the stimulation electrode can induce a global neuronal response with a PSTH > 1. More details about electrodes selection can be found in the Method section. We also have modified the figure legend to make it clearer: “Spontaneous activities for 10 minutes (gray period), specific stimulation electrodes selection for the patterns (green period), interval L pattern training (blue period), the X pattern training (pink period). ”

9. Fig.3: what is the different stimulation protocol used here?

**Response:** Thanks for your attention to this detail. The original statement may have caused a misunderstanding. The stimulation protocol used in Fig. 3 is the same as that in Fig. 1 and Fig. 2, where the neuronal network was trained using interval L and X stimulation protocols.

The purpose of Fig. 3 is to explore the functional network responses to the same stimulation patterns (same: interval L or X in the first and second rounds of training) versus different stimulation patterns (different: interval L vs. X in either the first or second round of training).

We have revised the figure legend to make it clearer. “ (Same: interval L or X in the first and second rounds of training; Different: interval L vs. X in either the first or second round of training).”

Reviewer #2 (Remarks to the Author):

The authors use cortical neural networks from rodents plated on multi-electrode arrays (MEAs). These networks are subjected to different electrical stimulation sequences, and electrophysiological activity is recorded before, during, and after the stimulation period. The main goals are to investigate these networks’ stimuli classification ability and the mechanisms involved. To perform this study, the authors mainly refer to connectivity analysis and network burst activity. Even though the manuscript presents very interesting results regarding the effects of external repeated stimuli on the network structure and the fact that different stimuli induce clearly different responses, at the current stage, it still needs clarifications and improvements. My main concern regards the number of experiments performed.

**Response:** We appreciate the positive feedback from the reviewer and have tried to improve our manuscript based on the nice suggestions. Particularly, we increase the number of cultures to 10. Our response is given in blue text, and changes/additions to the manuscript are provided in red.

Major comments:

1. I find the research intriguing, and the results on connectivity analysis are indeed in line with previous work aimed at investigating memory in-vitro. My main concern, though, is the number of cultures used for the final analysis. In each figure, the authors mention that n=5. Could the authors explain why such a low number of cultures was used? Because, in general, the number of cultures used in these studies, when on single MEAs (60 electrodes), is usually around 10 (see Dias et al. 2020, Lamberti et al. 2024), I am wondering if the authors performed any power analysis demonstrating that 5 cultures would be enough for statistical analysis interpretation.

**Response:** Thank you for this important suggestion. We fully agree with you about the concerns with culture numbers. We reviewed multiple studies that utilized low-density MEAs. While several studies had sample sizes of around five, the majority of cases reported culture numbers of approximately 10. Therefore, we have increased the cultures of 2 or 6 patterns recognition experiments to 10. After increasing the experimental sample size, our main conclusions regarding the pattern recognition capabilities of the biological neural network and the changes in functional networks remain unchanged. Following an increase in sample size, the classification accuracy for the six patterns continued to improve as the number of training days increased. In the comparative analysis of functional network structures induced by the same or different stimulus patterns, the results remained consistent with previous findings. It is important to note that, as shown in revised Figure 6a, the trend in changes to the evoked neural network structure after three days of training was no longer statistically significant, which contrasts with prior conclusions. However, this discrepancy is not attributable to the increased sample size, but rather may be a consequence of the re-construction of the functional network following spike sorting.

2. In their manuscript, one of the main findings is that after stimulation, not only do pre-existing connections change in strength but there is also the formation of new connections in the network (see line 155). How would the authors explain such a mechanism? In addition, although in methods, the authors mention the cultures that were used after 21 DIV, in the caption of Figure 1, the authors talk about cultures at DIV 14. Were the experiments performed at DIV 21 or DIV 14? I believe a clarification is needed. Cultures need 21 DIV to reach full maturation, so if experiments were performed before that, the creation of new connections might be due to the still ongoing network growth and not to the given stimulation.

**Response:** Thanks for your valuable comments**.** Our results indicate that training notably reshapes the functional connectivity of the cultured neural network, including new connections formation, which is similar to the study of speech recognition by brain organoids (*Cai et al., Nat Electron, 2023, 1032-1039*). It was also demonstrated that low-frequency stimulation could induce increased connections of the network in hippocampal cultures (*Gladkov et al., Sovremennye tehnologii v medicine 2017; 9(2): 61*). The mechanism behind the increase in network connectivity may be related to synaptic plasticity. It is known that short-term or long-term synaptic plasticity in neuronal networks can be observed under the induction of electrical activity. The biological mechanisms of synaptic plasticity can be explained through a series of biological processes driven by changes in gene expression. Synaptic plasticity is accompanied by structural plasticity in neurons, such as the remodeling of dendritic spines and the formation of new synapses(*Gellner et al., Brain Stimul. 2020;13(1):80-88.; Citri et al., Neuropsychopharmacol, 2008, 33, 18–41*). These structural changes are closely related to changes in gene expression and the activity of proteins, such as NMDA and AMPA receptors on the postsynaptic membrane. In terms of changes and regulation of gene expression, electrical stimulation can trigger the activation of various regulatory factors, such as CREB, which initiates multiple transcription factors and ultimately promotes the synthesis of various proteins, including synaptic-related proteins (*Flavell et al., Annu Rev Neurosci. 2008;31:563-90.*).

We agree about the 21 days needed for maturation. And, all experiments in this study were conducted using mature cultures after DIV 21. We previously used DIV 14 culture in Figure 1 primarily to better illustrate cell morphology. To avoid confusion, we have replaced the schematic picture with one from the culture at DIV 21.

3. As far as I understand, the authors use analysis on connectivity as the main readout. I would suggest already introducing in the introduction the importance of studying changes in connections to investigate memory and, consequently, information processing and storage by the networks. This will help with readability and will give input on why that was chosen as a metric.

**Response:** Thanks for your comment. We have revised the manuscript based on the your suggestion. We introduced the importance of studying connectivity changes in neural networks as it relates to memory, information processing, and storage.

“The study of changes in network connectivity has become a critical tool for investigating memory, information processing, and storage within neural networks. Alterations in the strength and patterns of synaptic connections are thought to underlie memory formation, with synaptic plasticity (such as long-term potentiation and depression) playing a key role in encoding, storing, and recalling information (23-25). Moreover, modifications in functional connectivity between neurons are crucial for understanding how neural networks process and retain information. Gaining insight into these changes enables researchers to explore how networks adapt to novel stimuli and consolidate memory (26-28). This makes the analysis of connectivity an important metric when investigating the underlying mechanisms of learning and memory in cultured neural networks, as it directly correlates with changes in network behavior and the ability to recognize and process patterns.”

4. In the data availability statement, the authors declare that no data were used for the study. What is meant by this? Weren’t recordings of neuronal activity performed and analyzed for the study? If yes, why are the authors not sharing data on an online data repository or upon request?

**Response:** We apologize for the mistake made by the author who submitted the manuscript. We have revised the manuscript and declare that the data supporting the main results of this work will be available upon publication.

“All electrophysiological data files and analysis code data are available from https://github.com/YunDid/Pattern-recognition/tree/master/pattern_recognition. Any additional information are available on request from the corresponding author.”

Minor comments:

1. In the methods section, under “Burst and network burst detection” in line 408, the authors wrote that if at least 20% of the channels were recording simultaneous activity, that was defined as a network burst. Can the authors better clarify why the threshold on the amount of electrodes was set to 20%? The definition and analysis of network bursts in MEAs is an ongoing debate; it will improve the strength of the manuscript by providing more details on how network bursts were identified, also given that it is one of the readouts used to interpret results.

**Response:** Thanks for your careful suggestion. We adopted the value of 20% by referring to previous work listed below. The 20% threshold we used is commonly found in the literature as a reasonable criterion for identifying a network burst.

Pasquale *et al.*: An evoked NB was detected when stimulation elicited a single-channel burst in at least 20% of active channels (similarly to what was done for detecting spontaneous events) (*Scientific Reports. 2017,7(1):9080*).

Bakkum *et al.*: a post-hoc criterion of a minimum number of activated channels per network burst was specified as 20% of the recording channels (*Front Comput Neurosci. 2014, 7:193.*).

Bologna *et al.*: the maximum inter-burst event interval for burst events within a network burst (maxIBeI) and secondly the minimum percentage of recording electrodes involved in a network burst (usually set at 20% the total number of active channels)(*Neural Netw. 2010, 23(6):685-97.* ).

Pasquale *et al.*: we intersected the resulting surface with the number of network bursts detected by our algorithm, setting the minimum percentage of electrodes involved (minPercElec) at 20%, which is the value we generally used to analyze our experiments (*J Comput Neurosci. 2010, 29(1-2):213-229.* ). Moreover this choice seems to be suitable for different experimental conditions, suggesting that 20% is approximately a constant threshold for the number of electrodes involved to give rise to a network event.

In conclusion, the 20% threshold was chosen based on its widespread use in the literature and its proven ability to provide reliable and consistent results for network burst detection.

2. In the methods section under “ Functional network construction and evaluation,” lines 446-447, the authors say that a widely used cross-correlation algorithm was adopted. Can the authors provide references to the works in which this algorithm was already used for analysis? In addition, in line 449, the authors refer to the spiking activity of 2 neurons. Were the authors applying any spike sorting algorithm? How were they capable of differentiating single neurons from the MEAs recording? In MEA (60 electrodes) recordings, electrodes acquire activity from groups of neurons and not single neurons, so if no spike sorting was applied probably it the sentence should change to “spike activity of 2 electrodes”.

**Response:** Thanks for your careful advices. We adopted the cross-correlation algorithm by referring to previous studies [*Ide et al., BMC Neurosci. 2007,8(2):63; Poliet al., Front Neural Circuits. 2015, 9:57.; Schroeter et al., J Neurosci. 2015, 35(14):5459-70.*]. We have cited relevant papers in our manuscript.

We fully agree with your opinion. In the original manuscript version, we did not use a spike sorting method. Therefore, the cross-correlation represented the relationship between the neural activity of two electrodes. However, based on the comments feedback from other reviewers, we have redone the data analysis when spiking sorting is needed. The results demonstrated that new analysis method did not change our conclusions, such as pattern recognition ability and neural network structure changes. In revised Figure 1e-g, Figure 2, Figure 3, Figure 4c-d, Figure 6, Figure 7 and supplementary figures (Figure S1, Figure S2, Figure S4 and Figure S5), spike sorting was adopted. Spike sorting processes are detailed in the Methods section.

3. The same goes for the Euclidean distances section; also, here, the authors say that it is a common method used to analyze changes in functional network structure but provide only one reference. Providing more references to similar work using the same method would strengthen the statement.

**Response:** Thank you for your comments. Dias *et al.* calculated the similarity between two functional matrices, before and after stimulation, using Euclidean distance, thereby demonstrating that low cholinergic activity promotes the formation and consolidation of memory (*Dias et al., Journal of neural engineering 18.4 (2021): 046051.*). Le et al. by calculating the Euclidean distance between these matrices, the study demonstrated how the network reorganizes, forming and consolidating memory traces. The approach also provided insights into the parallel storage of multiple memory traces in the network, as it showed that different stimuli lead to distinct changes in connectivity that do not interfere with one another (*le et al., Learning & memory. 22.12 (2015): 594-603.*).

4. References in lines 459 and 480 are in a different format compared to the rest of the manuscript.

**Response:** Thanks for your careful suggestion. The references have been updated to match the uniform format used throughout the manuscript.

5. Typo line 435 “… and The PSTH..”. The T in the should not be capitalized.

**Response:** Thanks for pointing out this mistake. We have fixed it.

Reviewer #3 (Remarks to the Author):

This is a study of how intelligence in pattern classification can arise from cultured neurons. This is a timely study that shows a new capability of intelligence to brain organoids. I found the study to be wonderful and have slight suggestions, some just out of curiosity.

**Response:** We appreciate the positive feedback from the reviewer and have tried to improve our manuscript based on the nice suggestions. Our response is given in blue text, and changes/additions to the manuscript are provided in red.

1. Another study that might be of interest in showing what brain organoids can do is this one: [https://academic.oup.com/pnasnexus/article/2/6/pgad188/7202378](https://academic.oup.com/pnasnexus/article/2/6/pgad188/7202378).

**Response:** Thank you for recommending the article by Joost et al. It is indeed a highly relevant and important study. Joost et al. provide an in-depth exploration of the prediction capabilities of cultured cortical neural networks for future stimuli and the roles of short-term and long-term memory in the prediction process. Specifically, they employed the mutual information (MI) framework to quantify the relationship between prediction and memory, offering a novel perspective on the functional properties of cultured neural networks.

We have already cited this citation in the discussion section of the revised manuscript and highlighted how its findings align with and complement our own research. This addition will enrich the context of our work and offer a more comprehensive understanding of the current developments and potential in the field of cultured neural networks.

2. Figure 1f: I'm not sure what the x and y axes are. Maybe put in caption?

**Response:** Thanks for your attention. In Figure 1f, the plot represents neuronal response strength to two different stimulation patterns, interval L and interval X. The x-axis indicates the number of spikes in response to the interval L stimulation pattern, while the y-axis indicates the number of spikes in response to the interval X stimulation pattern. We have modified the axis titles and figure legends: “The x-axis indicates the number of spikes in response to the interval L stimulation pattern, while the y-axis indicates the number of spikes in response to the interval X stimulation pattern.”

3. Before 2.2, maybe say you'll do more inputs? Supposedly a neural network has a memory of 1.4N/log N neurons or some such with the outer product rule, according to Hopfield's famous result-- we aren't coming close to that, but with that in mind, 2 seems somewhat unimpressive, but the results are actually impressive, so maybe just say that later on you do more patterns?

**Response:** Thank you for your insightful comment regarding the number of input patterns and for highlighting Hopfield's result on neural network memory capacity. We agree that the theoretical context you provided offers valuable insights, and we appreciate your suggestion to address the relatively small number of stimulation patterns in our study.

We have taken the nice suggestions and revised the manuscript.

“Our results demonstrated that the two stimulation patterns could be effectively classified based on the response properties of the cultured network after training. To establish a framework for validating the pattern classification capability of cultured neural networks, we initially selected two stimulation patterns. This approach allowed us to focus on investigating the fundamental mechanisms of structural and functional reorganization during training. Recognizing the need for a more comprehensive evaluation of the network's classification capabilities, we subsequently increased the number of stimulation patterns to explore the processing capacity and mechanisms of the biological neural network in response to more complex inputs.”

4. Line 150: Maybe try this method as well, or mention it?

<https://www.nature.com/articles/s41598-022-13674-4>

**Response:** We agree that this method is an interesting approach and we will be happy to try it in future work. In this manuscript, we have cited this paper and added relevant discussions: “While we have used the cross-correlation (CC) method to assess functional connectivity, future work will explore the use of Conditional Firing Probability (CFP) models to further characterize connectivity, particularly in detecting inhibitory connections and studying network dynamics during stimulation (69).”

5. Line 188: Maybe say that the parameter that's causing the tradeoff is the number of classes?

**Response:** Thank you for your valuable suggestion. You are correct that the tradeoff observed in our results is indeed influenced by the number of stimulation patterns. As the number of stimulation patterns increases, the classification task becomes more complex, leading to a decrease in the recognition accuracy of the neuronal network. We believe this reflects the inherent challenge for the network to differentiate between increasingly similar or complex input patterns. We have added the clarification: “The tradeoff in recognition accuracy is influenced by the number of stimulation patterns, as an increase in the number of classes introduces greater complexity to the classification task.”

6. Figure 4 just curious about how much better you can do with something more complicated than logistic regression.

**Response:** Thanks for your comments. We appreciate the suggestion, and tried SVM(rbf) and LDA for classification.

Data1

|  | SVM(rbf) | | | LDA | | | Logistic regression | | | |
| --- | --- | --- | --- | --- | --- | --- | --- | --- | --- | --- |
| pattern  numbers | 2 | 4 | 6 | 2 | 4 | 6 | 2 | 4 | 6 |  |
| Day1 | 0.97 | 0.83 | 0.79 | 0.97 | 0.90 | 0.84 | 0.99 | 0.90 | 0.83 |  |
| Day2 | 0.92 | 0.90 | 0.89 | 0.93 | 0.90 | 0.91 | 0.92 | 0.91 | 0.91 |  |
| Day3 | 0.95 | 0.88 | 0.88 | 0.96 | 0.91 | 0.93 | 0.98 | 0.92 | 0.90 |  |

Data2

|  | SVM(rbf) | | | LDA | | | Logistic regression | | | |
| --- | --- | --- | --- | --- | --- | --- | --- | --- | --- | --- |
| pattern  numbers | 2 | 4 | 6 | 2 | 4 | 6 | 2 | 4 | 6 |  |
| Day1 | 0.82 | 0.74 | 0.59 | 0.76 | 0.71 | 0.60 | 0.82 | 0.79 | 0.65 |  |
| Day2 | 0.95 | 0.81 | 0.64 | 0.88 | 0.79 | 0.63 | 0.95 | 0.85 | 0.69 |  |
| Day3 | 0.98 | 0.83 | 0.74 | 0.98 | 0.84 | 0.76 | 0.99 | 0.80 | 0.73 |  |

Data3

|  | SVM(rbf) | | | LDA | | | Logistic regression | | | |
| --- | --- | --- | --- | --- | --- | --- | --- | --- | --- | --- |
| pattern  numbers | 2 | 4 | 6 | 2 | 4 | 6 | 2 | 4 | 6 |  |
| Day1 | 0.90 | 0.73 | 0.56 | 0.88 | 0.76 | 0.60 | 0.91 | 0.77 | 0.62 |  |
| Day2 | 0.97 | 0.84 | 0.79 | 0.97 | 0.86 | 0.81 | 0.98 | 0.90 | 0.81 |  |
| Day3 | 0.94 | 0.89 | 0.79 | 0.98 | 0.92 | 0.85 | 0.97 | 0.93 | 0.84 |  |

The difference in performance between SVM, LDA, and Logistic Regression is generally small and doesn't show a dramatic increase in accuracy calculated by SVM(rbf).

7. Line 340: Cultured to cultured

**Response:** Thanks for your careful suggestion. We have fixed it.

8. Line 461: Can you talk about the pros and cons between your method and the method of Joost et al earlier?

**Response:** Thank you for raising this important point regarding the comparison between our method and the approach used in Joost et al.'s earlier study. We appreciate the opportunity to clarify the differences and provide insights into the advantages and limitations of each.

We have added the relevant discussions: “Joost et al. employed mutual information (MI) to quantitatively assess memory and prediction efficiency in neural networks, providing a detailed and mathematically rigorous framework for analyzing network dynamics (70). While their method is highly effective in measuring prediction accuracy, our study places greater emphasis on the relationship between changes in functional connectivity within the network and the subsequent improvements in recognition performance. In contrast to Joost et al., we did not explore the application of MI for evaluating memory. Nonetheless, their approach offers valuable insights into memory dynamics, and we plan to incorporate MI-based methods in future work to further investigate both memory and prediction aspects of our trained networks. ”
