# Supplementary material for: Repetitive training enhances the pattern recognition capability of cultured neural networks
Source: PLoS Comput Biol. 2025 Apr 22;21(4):e1013043. doi: 10.1371/journal.pcbi.1013043 (PMC12064202; doi:10.1371/journal.pcbi.1013043)
Supplement: Attachment — Submitted filename: point to point response.docx [file pcbi.1013043.s012.docx]

**Response to the reviewers’ comments**

Reviewer #2 :

1. In the discussion, line 363, the author now makes a comparison with a previous work from Lamberti et al. ( reference number 35 in the manuscript). In this work Lamberti et al explain the use of a novelle technique to analyze functional connectivity in neuronal networks based on maximum entropy models (MaxEnt). This new method was compared to a more commonly used one that is conditional firing probability (CFP). In this work it came out that MaxEnt is a valuable alternative that can also distinguish between excitatory and inhibitory connections, something that CFP can’t do because it mainly relies on excitatory connections. The author instead writes “…Conditional Firing Probability (CFP) models to further characterize connectivity, particularly in detecting inhibitory connections…” I believe they were referring to the MaxEnt method and not the CFP one. If this is not the case can the author clarify how CFP detects inhibitory connections?

**Response:** Thank you for pointing this out. We are sorry for our carelessness. In our original manuscript, we inadvertently attributed the advantage of detecting inhibitory connections to the CFP model instead of the MaxEnt framework. The study conducted by Lamberti et al. (Reference 35) demonstrates that the Maximum Entropy (MaxEnt) model can effectively distinguish between excitatory and inhibitory connections, whereas the Conditional Firing Probability (CFP) model, as a cross-correlation-based method, infers functional network connectivity through spontaneous activity patterns and characterizes the strengths and latencies of excitatory connections (as detailed in Reference 35).

We have revised the manuscript: While we have used the cross-correlation (CC) method to assess functional connectivity, future work will explore the use of Maximum Entropy (MaxEnt) models to estimate functional connectivity during stimulation and infer both excitatory and inhibitory connections (35).

2. In figure 1 line 136 the first sentence needs rephrasing. I believe that cultures were plated on MEAs at DIV 0 and used at DIV 21.

**Response:** Thank you for the comments. We apologize for any lack of clarity in our previous draft. In the binary classification results shown in Figure 1, the accuracy rate was already high on the first day of training (training day 1). As a result, the subsequent two days of training did not show further improvement in accuracy.

We have revised the manuscript to improve clarity: Likely due to the high accuracy achieved on the first day, further training did not significantly improve classification performance.
3. In the discussion line 399 and 404 the manuscript referenced ( number 41 in the reference list) has as first author Lamberti M. thus it should be referenced as Lamberti et al and not Joost et al.

Response: We sincerely appreciate the reviewer's careful reading and apologize for the error in citing the author's name. We have corrected it in the revised manuscript: Lamberti et al. employed mutual information (MI) to quantitatively assess memory and prediction efficiency in neural networks.

4. **Have the authors made all data and (if applicable) computational code underlying the findings in their manuscript fully available?**

The PLOS Data policy requires authors to make all data and code underlying the findings described in their manuscript fully available without restriction, with rare exception (please refer to the Data Availability Statement in the manuscript PDF file). The data and code should be provided as part of the manuscript or its supporting information, or deposited to a public repository. For example, in addition to summary statistics, the data points behind means, medians and variance measures should be available. If there are restrictions on publicly sharing data or code —e.g. participant privacy or use of data from a third party—those must be specified.

Reviewer #1: None

Reviewer #2: Yes

Reviewer #3: Yes

Response: All electrophysiological data files and analysis code data are available from <https://github.com/YunDid/Pattern-recognition/tree/master/pattern_recognition.> The screenshot below shows the file uploaded on github：
